# Glucose-lowering medications and glucose levels as the major determinants of progression of carotid atherosclerosis in middle-aged adults and elders: a community-based prospective study

**DOI:** 10.3389/fendo.2024.1425027

**Published:** 2024-10-18

**Authors:** Chao-Liang Chou, Shu-Xin Lu, Chun-Fang Cheng, Tzu-Wei Wu, Li-Yu Wang

**Affiliations:** ^1^ Department of Medicine, MacKay Medical College, New Taipei, Taiwan; ^2^ Department of Neurology, MacKay Memorial Hospital, New Taipei, Taiwan; ^3^ Tamsui Health Station, Department of Health, New Taipei City Government, New Taipei, Taiwan

**Keywords:** carotid atherosclerosis, carotid ultrasonography, diabetes mellitus, prospective study, glucose-lowering medications, disease progression

## Abstract

**Background:**

Few prospective studies explored the incidence and determinant of carotid atherosclerosis (CA) progression (CAP). This community-based prospective study focused on the effects of diabetes mellitus (DM) treatments and glucose levels on CAP risks.

**Methods:**

We followed up a group of 657 CA-positive middle-aged adults and elders for CAP. CAP was defined as an increase in the total number of carotid plaque and/or an increase in diameter stenosis by at least 10%.

**Results:**

After 4.05 years of followed-up, CAP was detected in 364 (55.4%) subjects. The multivariable-adjusted hazard ratios (HRs) were 1.805 (95% confidence interval [CI]: 1.374-2.358) and 0.694 (95% CI: 0.510-0.944) for elevated fasting plasma glucose (eFPG; FPG≥100 mg/dL) and glucose-lowering medications (GLM), respectively. As compared to GLM-negative+eFPG-positive subjects, the multivariable-adjusted HRs were 0.497 (95% CI: 0.373-0.662), 0.537(95% CI: 0.306-0.942), and 0.586 (95% CI: 0.412-0.833) for GLM-negative+eFPG-negative, GLM-positive+eFPG-negative, and GLM-positive+ eFPG-positive subjects, respectively. The multivariable-adjusted risks of CAP were similar between GLM-negative+eFPG-negative and GLM-positive+ eFPG-positive subjects (p=0.77). Stratified analyses showed that the multivariable-adjusted HRs per 5.0 mg/dL increase in FPG were significantly increased among GLM-negative subjects (HR=1.131; 95% CI: 1.094-1.171) and non-significantly decreased among GLM-positive subjects (HR=0.985; 95% CI: 0.957-1.013).

**Conclusion:**

We found that more than 50% of CA-positive subjects had CAP in 4 years and higher FPG significantly increased and GLM significantly decreased the risks of CAP. Additionally, GLM and FPG demonstrated an interactive effect on CAP risks. It seems possible that GLM may induce effects beyond lowering glucose levels and subsequently lowers CAP risks.

## 1 Introduction

Atherosclerosis is the progress of gradual constriction of arteries by plaque formation within the artery walls (1). Atherosclerotic diseases, including myocardial infarction and stroke, induce great impacts on global heath. In 2013, cardiovascular diseases (CVDs) accounted for 17.3 million deaths globally, which was 1.4-fold of that in 1990 (2). A recent report showed that the global CVD cases were 523 million in 2019, nearly 2-fold of that in 1990 (3). Moreover, CVD continues to be one of the major causes of disease burden. The global disability-adjusted life years (DALYs) caused by CVDs was 393 million DALYs in 2019, which was1.4-fold of that in 1990 (4). It was reported that aging of global population was the leading cause of significantly increased global mortality, morbidity, and burden (3, 4). Unfortunately, the number of aged population increase rapidly than before; it was 0.73 billion in 2020 and will be 1.30 billion in 2040 (5). Undoubtedly, health, social, and financial impacts caused by atherosclerotic diseases will be inevitable and enormous. Accordingly, formulating effective prevention measures is critically relevant and prompt.

Several prospective studies showed that carotid atherosclerosis (CA) is predictive to CVD risks (6). CA at the extracranial carotid arteries can be detected non-invasively and reliably by ultrasound in the fields. CA research may provide critical evidences for identifying target determinants and population for the prevention of atherosclerotic diseases. Several prevalence studies consistently showed that diabetes mellitus (DM) was one of the major modifiable determinants for the presence of CA (7–15). It is reasonable to assume that DM treatments may subsequently reduce the risks of atherosclerosis and CVDs. Indeed, a recent meta-analysis study, which included 30 clinical trials with 225305 DM patients, demonstrated that the risk of fatal and non-fatal atherosclerotic events for subjects who received glucose-lowering medications or strategies was significantly decreased (relative risk=0.92, 95% confidence interval [CI]: 0.89-0.95, p<0.0001) (16). A similar finding was reported by another independent meta-analysis study (17). Yet, reports on DM treatments and the incidence and progression of atherosclerosis are limited.

Additionally, dozens of prospective studies used ultrasounds to detect CA and followed-up the cohort members for CVD incidence (6). However, to our knowledge, only a very limited number of them reported the incidence and progression rates of CA (18–22). Prospective studies on DM with the risk of CA progression (CAP) were rare and inconsistent (19, 23–25). Moreover, there was no report concerning DM treatment with the incidence and determinant of CAP. As a result, we conducted this prospective community-based study on a group of CP-positive middle-aged and older adults to explore the incidence rate and determinants of CAP, with a primary focus on the effects of glucose level and DM treatments.

## 2 Materials and methods

### 2.1 Study subjects

**Figure 1:**
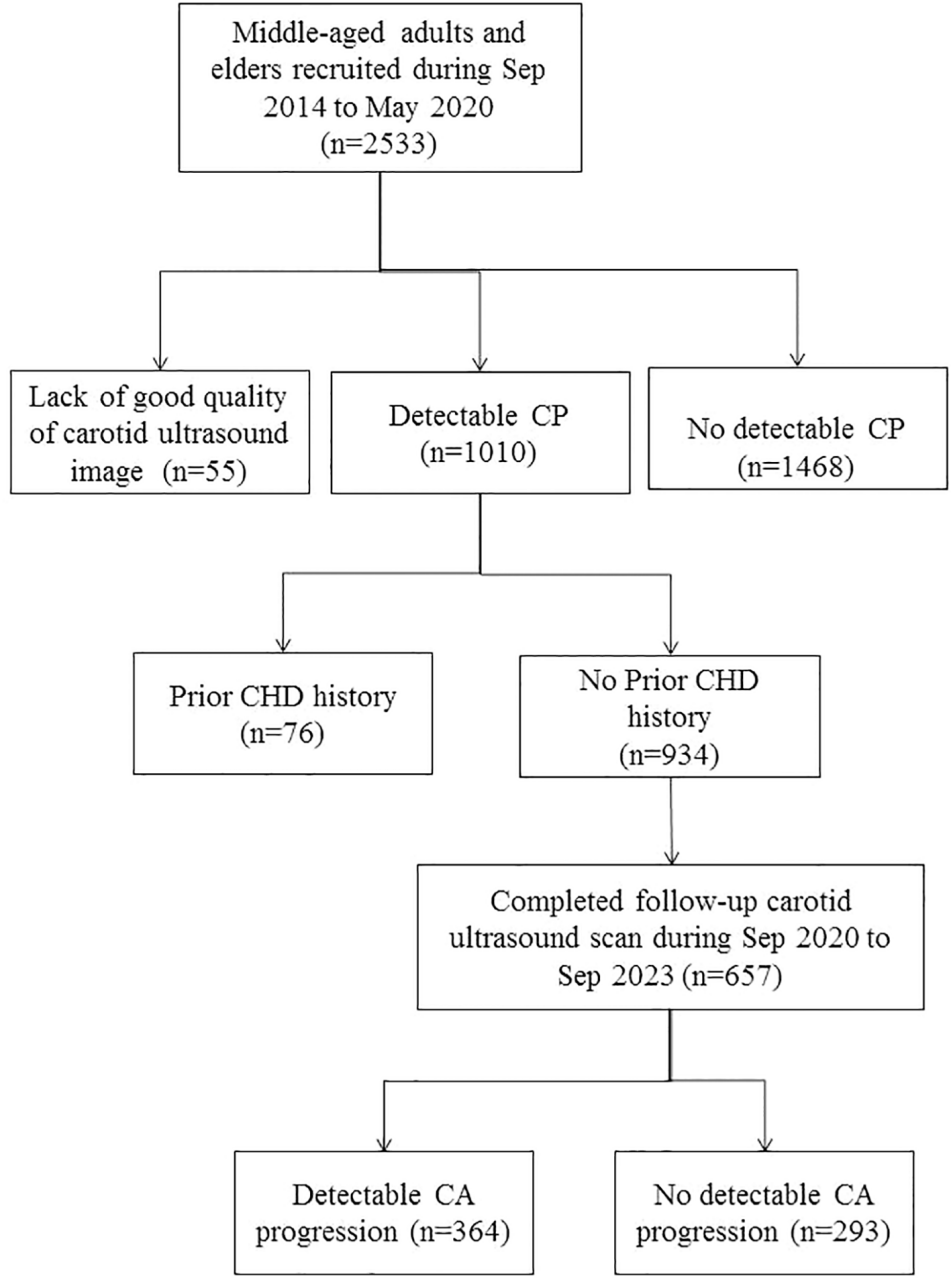
Steps of subject selection and categorization.

This is part of an ongoing community-based prospective study, which recruited community-dwelling middle-aged adults and elders from the northern coastal area of Taiwan (10, 13). From Sep 2014 to May 2020, well-informed invitation letters describing the objective and protocols of the study were sent to households with eligible subject(s). Residents who were willing to complete a structured questionnaire regarding personal health information and willing to provide blood samples were recruited. A total of 2533 residents aged 40-to-74 years voluntarily provided informed consent and were enrolled. Among 2478 subjects who had good quality of recorded carotid images, 1010 of them had detectable carotid plaque (CP) in their extracranial carotid arteries. Among CP-positive subjects, 76 who had a positive history of physician-diagnosed myocardial infarction or had ever received a cardiac catheter or stent were excluded, leaving a total of 934 subjects for follow-up for CAP (**Figure 1**).

The follow-up examinations were conducted from Sep 2020 to Sep 2023. Of the 934 CP-positive, 657 had completed follow-up carotid ultrasonography. The completion rate of follow-up was 70.3%. Among subjects who completed follow-up examinations, 364 subjects fulfilled the criteria of CAP (**Figure 1**). The study complied with the 1975 Helsinki Declaration on ethics in medical research and was reviewed and approved by the institutional review boards of MacKay Medical College (No. P990001; date of approval: 5 July 2010) and MacKay Memorial Hospital (No. 14MMHIS075; date of approval: 23 May 2014).

### 2.2 Measurements of anthropometric attributes and biochemical profiles

The measurements of anthropometric attributes and biochemical profiles have been described previously (13, 26). In brief, we used a digital system (BW-2200; NAGATA Scale Co. Ltd., Tainan, Taiwan) to measure the subject’s body weight (BWT) and body height (BHT). Waist circumference (WC) was measured at the level of mid-distance between the bottom of the rib cage and the top of the iliac crest. Hip circumference (HC) was the distance around the largest part of the subject’s hips. Body mass index (BMI) was used as the indicator of general obesity and calculated as body weight divided by (Body weight)^2^. Waist-to-hip ratio (WHR) and Waist-to-height ratio (WHtR) were used as the indicators of central obesity and were calculated as WC×100% divided by HC and BHT, respectively.

Blood pressure was measured three times, with an interval of 3 minutes, after 10 min of rest. The averages of repeated measurements of systolic blood pressure (SBP) and diastolic blood pressure (DBP) were used for analyses. The fasting blood levels of total cholesterol, high-density lipoprotein cholesterol (HDL-C), low-density lipoprotein cholesterol (LDL-C), triglycerides (FTG), and glucose (FPG) were determined by an autoanalyzer (Toshiba TBA c16000; Toshiba Medical System, Holliston, MA, USA) with commercial kits (Denka Seiken, Tokyo, Japan). In the study, non-HDL-C was calculated as (total cholesterol - HDL-C). Additionally, LDL- to HDL-C ratio (LHR) and non-HDL- to HDL-C ratio (nHHR) were calculated for each subject.

In the study, we used a structured questionnaire to collect data related to health behaviors and personal histories of common diseases. In the study, cigarette smoking and alcohol drinking were defined as having smoked cigarettes or drank alcohol-containing beverages at least four days per week during the past month before enrollment. DM was defined as subjects who fulfilled at least one of the criteria: (1) FPG≥126 mg/dL, (2) a positive history of physician-diagnosed DM, and (3) the use of glucose-lowering agents. Hypertension was defined as subjects who fulfilled at least one of the criteria: (1) SBP≥140 mm Hg or DBP ≥90 mm Hg, (2) a positive history of physician-diagnosed hypertension, and (3) a positive history of taking anti-hypertensive medications. Hyperlipidemia was defined as subjects who fulfilled at least one of the criteria: (1) TC≥240 mg/dL, LDL-C≥160 mg/dL, or FTG≥350 mg/dL; (2) a positive history of physician-diagnosed high blood lipids; and (3) a positive history of taking lipid-lowering medications.

### 2.3 Determination of carotid plaque

The measurements of carotid atherosclerosis had been described previously (10). In brief, we used a high-resolution digital color ultrasound system (GE Healthcare Logiq E; General Electric Company, Milwaukee, USA) to obtain the transverse and cross-sectional ultrasound images of the left and right extracranial carotid arteries, according to the protocol recommended by the American Society of Echocardiography (27). The baseline and follow-up carotid ultrasonography were performed by a well-trained and experienced technician who was blinded to subject’s clinical profile. All stored ultrasound images were retrieved and measured for CA by a well-trained and experienced research assistant who was also blinded to subject’s clinical profile at the central laboratory. The thickness between the lumen-intima and media-adventitia interfaces was measured blindly using built-in automatic contouring programs. In the study, a plaque was defined as a focal protrusion 50% greater than the surrounding vessel wall, an intima-media thickness (IMT) ≥1.5 mm, or local thickening ≥0.5 mm (28). The location and size of each carotid plaque were recorded. The degree of carotid stenosis was calculated as the percentage of maximal diameter reduction at carotid arteries as recommended by the European Carotid Surgery Trialists’ Collaborative Group (29).

### 2.4 Definition of CA progression

In the study, two criteria were used to define CA progression (CAP), including an increase in the total number of carotid plaque and a significant increase in the diameter stenosis. To evaluate the repeatability, we randomly selected 50 CP-positive subjects and re-measured their carotid images one month after the first measurement. A total of 153 CPs were identified from these CP-positive subjects at the first measurement and only 3 CPs were missed in the second measurement. The means (standard deviation [SD]) of diameter stenosis were 29.8% (12.3%) and 29.6% (12.7%) for the first and the second measurements, respectively. The SD of the differences in the diameter stenosis between the first and the second measurements of the same set of carotid images was 4.51%. Although the baseline and follow-up carotid ultrasound scans were performed by the same technician, it was possible that the angles and alignments between the probe and the blood vessels of the baseline and follow-up ultrasound scans might be slightly varied. Consequently, we used 10%, approximately 2.2-fold of the SD of the repeated CA measurements, as the cut-off value of measurement error. A significant increase in the diameter stenosis was defined as the diameter stenosis of the follow-up ultrasound scan is at least 10.0% larger than that of the baseline scan.

### 2.5 Statistical analyses

In the study, we calculated the mean (SD) and frequency distribution for continuous and categorical variables, respectively. The student’s t and the Pearson’s chi-square tests were used to test the significance of continuous and categorical variables between CAP-positive and –negative subjects. The Cochran-Armitage trend test was used to test the linear trend between age and cumulative incidence of CAP in female and male subjects. In addition to DM and glucose levels, all anthropometric and clinical factors showing significant differences between CAP-positive and –negative subjects were subject to association analyses. To assess the independent effects of DM treatment and glucose level, we used Cox proportional-hazard regression analyses to estimate their hazard ratios (HRs) of CAP and controlled for the potential confounding effects of other cardio-metabolic factors. Age and sex were forced in the regression model to control for their effects. The other cardio-metabolic factors which showed significant correlations with the risk of CAP after controlling for the effects of age and sex were included in multi-variable regression anlyses. We used stepwise selection method to obtain the best-fit model. The criteria for inclusion into and stay in the regression model were 0.05 and 0.1, respectively. All statistical analyses were performed using SAS 9.4 (SAS Institute Inc., Cary, NC, USA).

## 3 Results

### 3.1 Baseline clinical characteristics of study subjects

Among 934 CP-positive subjects who had no prior CVD history, 657 (70.3%) subjects had completed follow-up examinations (**Figure 1**). The mean (SD) interval between the baseline and the follow-up examinations was 4.05 (0.73) years.

Among subjects who completed follow-up examinations, 274 (41.7%) of them were male, and the mean (SD) age at enrollment was 61.8 (6.8) years. **Table 1** also shows that the prevalence rates of hypertension, hyperlipidemia, and DM were 52.7%, 54.6%, and 19.5%, respectively. More than 1/3 of the subjects had received anti-hypertensive or lipid-lowering medications, and 18.4% of the subjects had received glucose-lowering medications.

**Table 1:** Baseline characteristics of 657 CP-positive middle-aged adults and elders.

	All (n=657)		CAP-negative (n=293)		CAP-positive (n=364)		p-value
	Mean	(SD)	Mean	(SD)	Mean	(SD)	
Age at enrollment (years)	61.8	(6.8)	60.4	(7.1)	63.0	(6.3)	<0.0001
BMI (kg/m^2^)	24.7	(3.4)	24.5	(3.6)	24.9	(3.3)	0.19
WC (cm)	89.1	(8.9)	88.6	(9.1)	89.5	(8.8)	0.22
HC (cm)	97.1	(6.7)	97.3	(7.1)	96.9	(6.4)	0.50
WHR (%)	91.7	(6.0)	91.0	(5.8)	92.2	(6.1)	0.0084
WHtR (%)	55.6	(5.7)	55.4	(5.8)	55.7	(5.6)	0.44
SBP (mm Hg)	129.2	(16.9)	127.3	(16.5)	130.7	(17.1)	0.011
DBP (mm Hg)	75.7	(10.7)	75.1	(10.8)	76.1	(10.6)	0.25
TC (mg/dL)	204.7	(41.4)	204.2	(39.3)	205.1	(43.1)	0.78
LDL-C (mg/dL)	122.4	(34.2)	121.0	(32.4)	123.5	(35.6)	0.34
HDL-C (mg/dL)	54.5	(14.0)	55.9	(14.3)	53.4	(13.7)	0.027
Non-HDL-C (mg/dL)	15.0	(3.9)	14.8	(3.7)	15.2	(4.0)	0.27
LHR	2.37	(0.82)	2.28	(0.77)	2.43	(0.85)	0.018
nHHR	2.93	(1.07)	2.82	(1.00)	3.02	(1.12)	0.019
Log (FTG, mg/dL)	4.65	(0.51)	4.63	(0.51)	4.67	(0.52)	0.33
FPG (mg/dL)	98.2	(28.4)	96.3	(23.1)	99.8	(32.1)	0.11
	n	(%)	n	(%)	n	(%)	
Male sex	274	(41.7)	102	(34.8)	172	(47.3)	0.0017
Schooling years≤12 years	218	(33.2)	94	(32.1)	124	(34.1)	0.65
Cigarette smoking	148	(22.5)	48	(16.4)	100	(27.5)	0.0010
Alcohol drinking	97	(14.8)	30	(10.2)	67	(18.4)	0.0048
BMI≥27.0 kg/m^2^	156	(23.7)	65	(22.1)	91	(25.0)	0.45
Hypertension	346	(52.7)	138	(47.1)	208	(57.1)	0.013
Hyperlipidemia	359	(54.6)	145	(49.5)	214	(58.8)	0.021
Diabetes mellitus	128	(19.5)	45	(15.4)	83	(22.8)	0.022
Antihypertensive medications	262	(39.9)	101	(34.5)	161	(44.2)	0.014
Lipid-lowering medications	245	(37.3)	103	(35.2)	142	(39.0)	0.35
Glucose-lowering medications	121	(18.4)	42	(14.3	79	(21.7)	0.020

BMI, body mass index; CAP, progression of carotid atherosclerosis; FPG, fasting plasma glucose; FTG, fasting triglycerides; HC, hip circumference; LHR, LDL- to HDL-C ratio; nHHR, non-HDL-C to HDL-C ratio; WC, waist circumference WHR, waist-to-hip ratio; WHtR, waist-to-height ratio.

**Figure 2:**
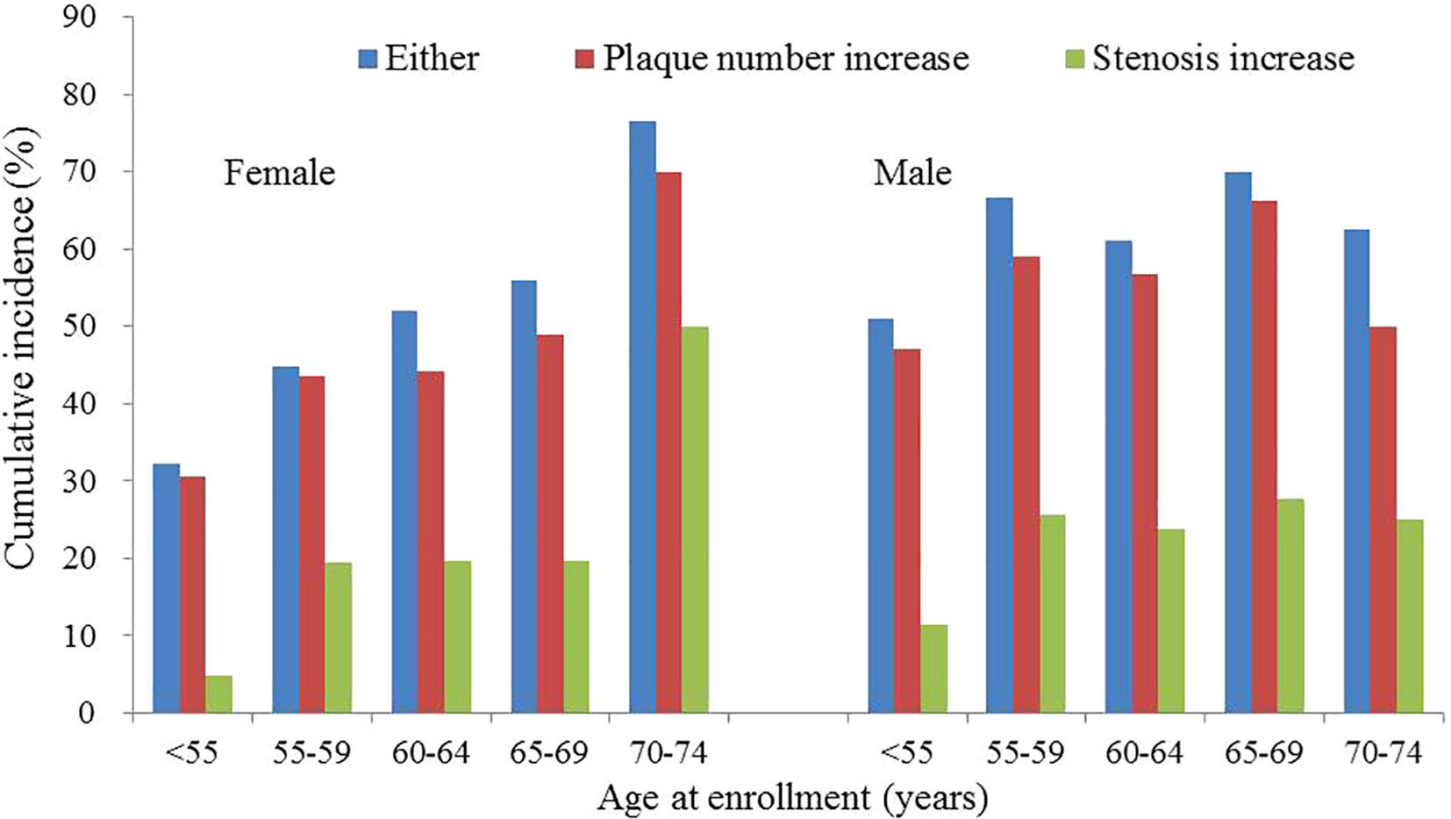
Progression rates of carotid atherosclerosis in male and female subjects.

A total of 192 females and 172 males had CAP. The corresponding cumulative progression rates were 50.1% (95% CI: 43.0%-57.3%) and 62.8% (95% CI: 53.4%-72.2%) for females and males, respectively. Among female subjects, the cumulative progression rates were approximately 1/3 for subjects aged <55 years, more than 1/2 for subjects aged 60-64 years, and greater than 3/4 for subjects aged 70 years or older (**Figure 2**, **Supplementary Table**). Among male subjects, the cumulative progression rates were approximately 1/2 for subjects aged <55 years and ranged from 60% to 70% for subjects aged 55 years or older (**Figure 2**, **Supplementary Table**). The linear trend between age and cumulative progression rate was significant in female subjects (p-value of trend test <0.0001) and non-significant in male subjects (p-value of trend test=0.11).

### 3.2 Comparisons of baseline clinical characteristics between subjects with and without incident atherosclerosis

**Table 1** also shows that compared to CAP-negative subjects, CAP-positive subjects had significantly higher means of age at enrollment, WHR, SBP, LHR, and nHHR. The mean HDL-C of CAP-positive subjects was significantly lower than that of CAP-negative subjects. The proportions of male sex, cigarette smoking, alcohol drinking, hypertension, hyperlipidemia, and DM of CAP-positive subjects were significantly higher than those of CAP-negative subjects. As compared to CAP-negative subjects, the proportions of having received anti-hypertensive and glucose-lowering medications were also significantly higher in CAP-positive subjects. There was no significant difference for other baseline clinical characteristics between CAP-positive and -negative subjects.

### 3.3 Association analyses for risk of carotid atherosclerosis

Univariable Cox regression analyses showed that the risks of CAP were significantly correlated with older age, male sex, higher WHR, and increased levels of LHR, nHHR, and FPG. The HRs of CAP were of borderline significance for cigarette smoking, alcohol drinking, hypertension, and hyperlipidemia (**Table 2**). The HRs of CAP were non-significant for DM, glucose-lowering medications, and other factors. After controlling for the effects of age and sex, the adjusted risks of CAP remained significantly increased for higher levels of WHR, LHR, nHHR, and FPG. The age-sex-adjusted HR was 1.253 (95% CI: 1.148-1.367; p<0.0001) for per 5.0% increase in WHR and were 1.223 (95% CI: 1.079-1.387; p=0.0017) and 1.127 (95% CI: 1.024-1.241; p=0.015) for per 1.0 increase in LHR and nHHR, respectively. The age-sex-adjusted HR was 1.023 (95% CI: 1.009-1.036; p=0.0008) for per 5.0 mg/dL increase of FPG.

**Table 2:** Analyses for progression of carotid plaque with baseline characteristics in middle-aged adults and elders.

	Univariable		Age-sex-adjusted	
	HR	(95% CI)	HR^1^	(95% CI)
Age, per 5.0 years	1.126^**^	(1.039-1.220)	1.116^*^	(1.029-1.208)
Sex (M/F)	1.355^**^	(1.102-1.666)	1.324^*^	(1.076-1.629)
WHR, per 5.0%	1.282^**^	(1.178-1.395)	1.253^**^	(1.148-1.367)
SBP, per 5.0 mm Hg	1.025	(0.995-1.055)	1.020	(0.990-1.051)
HDL-C, per 5.0 mg/dL	0.971	(0.934-1.009)	0.984	(0.943-1.026)
LHR, per 1.0	1.203^**^	(1.065-1.359)	1.223^**^	(1.079-1.387)
nHHR, per 1.0	1.119^*^	(1.020-1.229)	1.127^*^	(1.024-1.241)
FPG, per 5.0 mg/dL	1.024^**^	(1.010-1.037)	1.023^**^	(1.009-1.036)
Cigarette smoking (Y/N)	1.248^+^	(0.988-1.576)	1.073	(0.819-1.406)
Alcohol drinking (Y/N)	1.278^+^	(0.978-1.670)	1.087	(0.819-1.443)
Hypertension (Y/N)	1.194^+^	(0.969-1.470)	1.135	(0.920-1.400)
Hyperlipidemia (Y/N)	1.193^+^	(0.967-1.471)	1.228^+^	(0.995-1.516)
Diabetes mellitus (Y/N)	1.181	(0.923-1.510)	1.174	(0.917-1.502)
Anti-hypertensive medications (Y/N)	1.116	(0.907-1.373)	1.054	(0.855-1.300)
Glucose-lowering medications (Y/N)	1.133	(0.881-1.456)	1.126	(0.876-1.447)

^1^HRs for age and sex were obtained from the model containing age and sex.

CI, confidence interval; FPG, fasting plasma glucose; HR, hazard ratio; LHR, LDL- to HDL-C ratio; nHHR, non-HDL-C to HDL-C ratio; WHR, waist-to-hip ratio.

^+^, 0.05<p<0.10; ^*^, 0.005<p<0.05; ^**^, 0.0001<p<0.005; ^***^, p<0.0005.

### 3.4 Multivariable analyses for risk of carotid atherosclerosis progression

Models I to II showed that risks of CAP were significantly increased for subjects with higher LHR and WHR (**Table 3**). The multivariable-adjusted HR for per 1.0 increase in LHR was approximately 1.15 and was approximately 1.23 for per 5.0% increase in WHR. Model I also showed that as compared to subjects who had a normal FPG level, the multivariable-adjusted HR of CAP was 1.802 (95% CI: 1.374-2.358; p<0.0001) for subjects who had an elevated FPG level (≥100 mg/dL). The multivariable-adjusted HR of CAP was 0.694 (95% CI: 0.510-0.944; p=0.020) for subjects with a positive history of glucose-lowering medications compared to those without.

**Table 3:** Multivariable Cox proportional-hazard models for the progression of carotid atherosclerosis in middle-aged adults and elders.

Variable			HR^1^	(95% CI)
			Model I	
LHR, per 1.0			1.151^*^	(1.012-1.310)
WHR, per 5.0%			1.227^***^	(1.123-1.340)
Elevated FPG (≥100/<100 mg/dL)			1.802^***^	(1.374-2.358)
Glucose-lowering medications (Y/N)			0.694^*^	(0.510-0.944)
			Model II	
LHR, per 1.0			1.145^*^	(1.006-1.303)
WHR, per 5.0%			1.229^***^	(1.124-1.343)
Group	Glucose-lowering medications	FPG (mg/dL)		
1	No	≥100	1.000	
2	No	<100	0.497^***^	(0.373-0.662)
3	Yes	<100	0.537^*^	(0.306-0.942)
4	Yes	≥100	0.586^**^	(0.412-0.833)

^1^HRs were also adjusted for age and sex.

CI, confidence interval; FPG, fasting plasma glucose; HR, hazard ratio; LHR, LDL- to HDL-C ratio; WHR, waist-to-hip ratio.

^*^, 0.005<p<0.05; ^**^, 0.0001<p<0.005; ^***^, p<0.0005.

To evaluate the combined effects of glucose-lowering medications and FPG level, subjects were categorized into four groups. Model II showed that as compared to Group1, the multivariable-adjusted risks of CAP were significantly decreased for Group 2 (HR=0.497; 95% CI: 0.373-0.662; p<0.0001), Group 3 (HR=0.537; 95% CI: 0.306-0.942; p=0.030), and Group 4 (HR=0.586; 95% CI: 0.412-0.833; p=0.0029). Compared to Group 2, the multivariable-adjusted risk of CAP only slightly increased for Group 4 (HR=1.178; 95% CI=0.884-1.569; p=0.26).

### 3.5 Stratified analyses for risk of carotid atherosclerosis progression

Among subjects who had no history of glucose-lowering medications, significantly increased risks of CAP were correlated with increased levels of WHR and FPG (**Table 4**). The multivariable-adjusted HR was 1.169 (95% CI: 1.059-1.291; p=0.019) for per 5.0% decrease in WHR and was 1.131 (95% CI: 1.094-1.171; p<0.0001) for per 5.0 mg/dL increase in FPG. Among subjects who had a positive history of glucose-lowering medications, the risks of CAP were significantly increased for higher WHR (HR=1.431; 95% CI: 1.160-1.766; p=0.0008) and non-significantly slightly increased for higher FPG level.

**Table 4:** Multivariable Cox proportional-hazard models for the progression of carotid atherosclerosis in middle-aged adults and elders.

Variable	All subjects		Subjects without glucose-lowering medications		Subjects with glucose-lowering medications	
	HR^1^	(95% CI)	HR^1^	(95% CI)	HR^1^	(95% CI)
LHR, per 1.0	1.159^*^	(1.019-1.319)	1.139^+^	(0.983-1.319)	1.319^+^	(0.989-1.760)
WHR, per 5.0%	1.232^***^	(1.128-1.345)	1.169^**^	(1.059-1.291)	1.431^**^	(1.160-1.766)
FPG, per 5.0 mg/dL	1.024^*^	(1.007-1.046)	1.131^***^	(1.094-1.171)	1.015	(0.987-1.044)
Glucose-lowering medications (Y/N)	0.785	(0.566-1.090)	–		–	

^1^HRs were also adjusted for age and sex.

CI, confidence interval; FPG, fasting plasma glucose; HR, hazard ratio; LHR, LDL- to HDL-C ratio; WHR, waist-to-hip ratio.

^+^, 0.05<p<0.10; ^*^, 0.005<p<0.05; ^**^, 0.0001<p<0.005; ^***^, p<0.0005; -, not included.

## 4 Discussion

In this community-based study, we followed up a group of 657 CP-positive middle-aged and older adult subjects for CAP. We found that greater than one-half of the cohort members had CAP. Multivariable-adjusted risks of CAP were significantly elevated for increased glucose levels and significantly decreased for glucose-lowering medications. Additionally, we found a significant interactive effect on the CAP risk between glucose-lowering medications and FPG levels. To our knowledge, only a very limited number of community- or population-based prospective studies had explored the incidence and determinant of CAP.

In this community-based study, after 4 years of follow-up, CAP was detected in 364 (55.4%) subjects. Although there were community- or population-based studies that used ultrasounds to determine the degree of CA, only a very limited number of them reported the progression of preclinical atherosclerosis (19, 20, 25). Additionally, there is a significant variation in the definitions of CAP among studies. The definitions of atherosclerosis progression included changes in the total plaque area (on a continuous scale) (18), development of new plaque (19, 30), increase in plaque score (24), and increase in total plaque volume (31). Additionally, some studies used single criteria and others used multiple criteria or modalities to define atherosclerosis progression. A single criterion was used in the Tromsø Study (18), the MESA study (19), a recent Italian study (24), the CCCC study (30), and a recent Chinese study (31). The present study and the Reykjavik REFINE-study (20) used two criteria to define atherosclerosis progression. The PESA study used multiple imaging instruments, including 2-D and 3-D ultrasonography and CT, and findings of multi-territory of blood vessels to define atherosclerosis progression (25). As a result, the lack of established criteria for defining CAP results in difficulty in comparing progression rates among studies. It seems necessary to establish an evidence-based indicator of atherosclerosis progression.

In this community-based study, two criteria were used to define atherosclerosis progression, including an increase in the total number of carotid plaque and a significant increase in the diameter stenosis. Undoubtedly, an increase in the total number of carotid plaque is a valid indicator of atherosclerosis progression and has been used in several recent studies (19, 20, 30). However, no report used ‘significant increase in the diameter stenosis’. In this study, we considered the influences of measurement errors and considered it as a valid indicator of CAP. There are two sources of measurement errors in determining the severity of atherosclerosis, including variation in the images obtained during ultrasound scans and variation in measuring the same images. In this study, the ultrasound system was operated by the same well-trained and experienced technician and the degrees of diameter stenosis of all recorded images were determined in the central laboratory by the same research assistant. Accordingly, the only source of measurement error in this study was the intra-observer variation. To evaluate the magnitude of intra-observer variation in CA measurements, we selected a random sample of CP-positive subjects and re-measured the degree of diameter stenosis blindly one month after the first measurement. Only 2.0% of CPs detected in the first measurement was missed in the second measurement. The SD of the differences in the diameter stenosis of the repeated measurements was 4.51%. Furthermore, we considered that intra-observer variation in the carotid images obtained from the baseline and follow-up ultrasound scans may emerge. Therefore, this study used an increase in diameter stenosis by at least 10%, which is 2.22-fold of the SD of repeated measurements, as the cut-off value of measurement error.

In this study, we found that after controlling for the effects of age and sex, risks of CAP were significantly correlated with some modifiable risk factors, including WHR, glucose, and LHR. Previous prospective studies also demonstrated the critical roles of modifiable risk factors in atherosclerosis progression, yet the significant determinants varied among studies. In the Tromos study, an increase in total plaque area was correlated with higher total cholesterol levels, higher SBP, cigarette smoking (18) and abdominal adiposity (21). In the MESA study, elevated levels of total cholesterol, glucose, and SBP, cigarette smoking, and decreased levels of HDL-C were correlated with significantly higher risks of the formation of carotid plaque (19). The PESA study showed that dyslipidemia, smoking, and family history of CVD were significantly independent predictors of atherosclerosis progression in subjects who had atherosclerosis at baseline (25). More recently, the subsequent follow-up of the PESA study showed that active smoking, higher LDL-C and SBP, and global plaque volume were correlated with significantly higher age-sex-adjusted risks of peripheral subclinical atherosclerosis disease progression (22). The difference in the significant determinants of atherosclerosis progression among studies may probably be explained by the different definitions and instruments used to detect atherosclerosis progression. It is also possible that arterial plaque at different territories of blood vessels and different indicators of atherosclerosis progression represent different entities or pathogenesis of atherosclerotic diseases. To test our speculation, large prospective studies that use multiple instruments to assess the atherosclerosis at different territory of blood vessels are necessary.

Our and several previous cross-sectional studies showed that DM was significantly correlated with higher likelihoods of having CP (7–15). However, prospective studies were rare and depicted inconsistent findings. The risk of atherosclerosis progression for DM was significantly increased in the NEFRONA study (23) but was non-significantly slightly increased in others (19, 24, 25). In this study, we also found that CAP risks were not significantly different between DM patients and non-DM controls. However, stratified analyses showed inequitable effects of glucose levels on CAP risks. The multivariable-adjusted HR of CAP for increased FPG level was close to the null value in subjects with a positive history of glucose-lowering medications, but it was significantly increased in subjects without a history of glucose-lowering medications. It is likely that the inconsistent findings from previous prospective studies may be attributable to the confounding effects of glucose-lowering medications.

In addition, we found a significant interactive effect between glucose-lowering medications and glucose level on CAP risk. As compared to subjects who had no history of glucose-lowering medications and an elevated FPG level (**Table 3**; Group 1), the multivariable-adjusted risks of CAP significantly decreased by more than 50% for subjects who had no history of glucose-lowering medications and a normal FPG level (Group 2); and, decreased by more than 40% for subjects who had a history of glucose-lowering medications, regardless of their FPG levels (Group 3 & 4). The multivariable-adjusted risks of CAP were similar for subjects who had no history of glucose-lowering medications and a normal FPG level (Group 2) and subjects who had a history of glucose-lowering medications and an elevated FPG level (Group 4). Although no report correlates glucose-lowering medications with CAP, there were clinical trials on DM patients showing anti-hyperglycemic therapies significantly reduce the risks of major adverse cardiovascular outcomes (16, 17). We hypothesize that glucose-lowering medications may induce beneficial effects beyond lowering glucose level and subsequently reduce the risk of CAP and atherosclerotic diseases. Our speculation was supported by two lines of evidence. The first, DM treatments were correlated with significant improvement in several cardiovascular risk factors, including blood pressure, HDL-C, and high-sensitivity C-reactive protein (32). The second, DM treatments reduce glucose variability. A recent follow-up study showed that increased glucose variability was correlated with higher risks of atherosclerosis progression (31). More large prospective studies are necessary to support our hypothesis further.

This study has two potential limitations. The observational nature of the present study means that causal inference must be made with caution. In addition, we found that glucose-lowering medications significantly lower the risk of CAP. However, most of the subjects were not able to provide the name and type of their prescribed drugs. As a result, we were not able to carry out stratified analyses to estimate the effects of individual agent.

In conclusion, this study found that higher glucose level significantly increased and glucose-lowering medications significantly decreased the risks of atherosclerosis progression. Moreover, there was an interactive effect between glucose level and glucose-lowering medications on the risk of CAP. Among subjects with a history of glucose-lowering medications, the multivariable-adjusted risk of CAP was non-significantly slightly increased for elevated glucose level. However, among subjects without a history of glucose-lowering medications, the multivariable-adjusted risk of CAP was significantly increased for elevated glucose level. It is likely that glucose-lowering medications may induce effects beyond lowering glucose level. The underlying mechanism warrants further exploration.

## Data Availability

The raw data supporting the conclusions of this article will be made available by the authors, without undue reservation.

## References

[1] Bentzon JF Otsuka F Virmani R Falk E. Mechanisms of plaque formation and rupture. Circ Res. (2014) 114:1852–66. doi: 10.1161/CIRCRESAHA.114.302721 24902970

[2] Roth GA Forouzanfar MH Moran AE Barber R Nguyen G Feigin VL. Demographic and epidemiologic drivers of global cardiovascular mortality. N Engl J Med. (2015) 372:1333–41. doi: 10.1056/NEJMoa1406656 PMC4482354 25830423

[3] Roth GA Mensah GA Johnson CO Addolorato G Ammirati E Baddour LM. Global burden of cardiovascular diseases and risk factors, 1990-2019: Update From the GBD 2019 Study. J Am Coll Cardiol. (2020) 76:2982–3021. doi: 10.1016/j.jacc.2020.11.010 33309175 PMC7755038

[4] GBD. 2019 Diseases and Injuries Collaborators. Global burden of 369 diseases and injuries in 204 countries and territories, 1990-2019: a systematic analysis for the Global Burden of Disease Study 2019. Lancet. (2020) 396:1204–22. doi: 10.1016/S0140-6736(20)30925-9 PMC7567026 33069326

[5] The United Nations. Department of Economics and Social Affairs, The United Nations. In: World Population Prospects. (New York, USA: United Nations) (2019). Available at: http://population.un.org/wpp/.

[6] Naqvi TZ Lee MS. Carotid intima-media thickness and plaque in cardiovascular risk assessment. JACC Cardiovasc Imaging. (2014) 7:1025–38. doi: 10.1016/j.jcmg.2013.11.014 25051948

[7] Mostaza JM Lahoz C Salinero-Fort MA de Burgos-Lunar C Laguna F Estirado E. Carotid atherosclerosis severity in relation to glycemic status: a cross-sectional population study. Atherosclerosis. (2015) 242:377–82. doi: 10.1016/j.atherosclerosis.2015.07.028 26275375

[8] Woo SY Joh JH Han SA Park HC. Prevalence and risk factors for atherosclerotic carotid stenosis and plaque: a population-based screening study. Med (Baltimore). (2017) 96:e5999. doi: 10.1097/MD.0000000000005999 PMC5287981 28121957

[9] Wang X Li W Song F Wang L Fu Q Cao S. Carotid atherosclerosis detected by ultrasonography: a national cross-sectional study. J Am Heart Assoc. (2018) 7:e008701. doi: 10.1161/JAHA.118.008701 29622590 PMC6015437

[10] Chou CL Wu YJ Hung CL Liu CC Wang SD Wu TW. Segment-specific prevalence of carotid artery plaque and stenosis in middle-aged adults and elders in Taiwan: a community-based study. J Formos Med Assoc. (2019) 118:64–71. doi: 10.1016/j.jfma.2018.01.009 29395388

[11] Poorthuis MHF Halliday A Massa MS Sherliker P Clack R Morris DR. Validation of risk prediction models to detect asymptomatic carotid stenosis. J Am Heart Assoc. (2020) 9:e014766. doi: 10.1161/JAHA.119.014766 32310014 PMC7428515

[12] Song P Fang Z Wang H Cai Y Rahimi K Zhu Y. Global and regional prevalence, burden, and risk factors for carotid atherosclerosis: a systematic review, meta-analysis, and modelling study. Lancet Glob Health. (2020) 8:e721–9. doi: 10.1016/S2214-109X(20)30117-0 32353319

[13] Wu TW Chou CL Cheng CF Lu SX Wang LY. Prevalences of diabetes mellitus and carotid atherosclerosis and their relationships in middle-aged adults and elders: a community-based study. J Formos Med Assoc. (2022) 121:1133–40. doi: 10.1016/j.jfma.2021.10.005 34674902

[14] Yang D Iyer S Gardener H Della-Morte D Crisby M Dong C. Cigarette smoking and carotid plaque echodensity in the northern Manhattan study. Cerebrovasc Dis. (2015) 40:136–43. doi: 10.1159/000434761 PMC4567425 26227885

[15] Wu TW Chou CL Cheng CF Lu SX Wu YJ Wang LY. Associations of genetic markers of diabetes mellitus with carotid atherosclerosis: a community-based case-control study. Cardiovasc Diabetol. (2023) 22:51. doi: 10.1186/s12933-023-01787-7 36894991 PMC9999522

[16] Ghosh-Swaby OR Goodman SG Leiter LA Cheng A Connelly KA Fitchett D. Glucose-lowering drugs or strategies, atherosclerotic cardiovascular events, and heart failure in people with or at risk of type 2 diabetes: an updated systematic review and meta-analysis of randomized cardiovascular outcome trials. Lancet Diabetes Endocrinol. (2020) 8:418–35. doi: 10.1016/S2213-8587(20)30038-3 32333878

[17] Hasebe M Yoshiji S Keidai Y Minamino H Murakami T Tanaka D. Efficacy of antihyperglycemic therapies on cardiovascular and heart failure outcomes: an updated meta-analysis and meta-regression analysis of 35 randomized cardiovascular outcome trials. Cardiovasc Diabetol. (2023) 22:62. doi: 10.1186/s12933-023-01773-z 36935489 PMC10024854

[18] Herder M Johnsen SH Arntzen KA Mathiesen EB. Risk factors for progression of carotid intima-media thickness and total plaque area: a 13-year follow-up study: the Tromsø Study. Stroke. (2012) 43:1818–23. doi: 10.1161/STROKEAHA.111.646596 22550052

[19] Tattersall MC Gassett A Korcarz CE Gepner AD Kaufman JD Liu KJ. Predictors of carotid thickness and plaque progression during a decade: the Multi-Ethnic Study of Atherosclerosis. Stroke. (2014) 45:3257–62. doi: 10.1161/STROKEAHA.114.005669 PMC4213289 25213342

[20] Sturlaugsdottir R Aspelund T Bjornsdottir G Sigurdsson S Thorsson B Eiriksdottir G. Predictors of carotid plaque progression over a 4-year follow-up in the Reykjavik REFINE-study. Atherosclerosis. (2018) 269:57–62. doi: 10.1016/j.atherosclerosis.2017.12.005 29274849

[21] Imahori Y Mathiesen EB Morgan KE Frost C Hughes AD Hopstock LA. The association between anthropometric measures of adiposity and the progression of carotid atherosclerosis. BMC Cardiovasc Disord. (2020) 20:138. doi: 10.1186/s12872-020-01417-0 32183704 PMC7079386

[22] Mendieta G Pocock S Mass V Moreno A Owen R García-Lunar I. Determinants of progression and regression of subclinical atherosclerosis over 6 years. J Am Coll Cardiol. (2023) 82:2069–83. doi: 10.1016/j.jacc.2023.09.814 37993199

[23] Palanca A Castelblanco E Perpiñán H Betriu À Soldevila B Valdivielso JM. Prevalence and progression of subclinical atherosclerosis in patients with chronic kidney disease and diabetes. Atherosclerosis. (2018) 276:50–7. doi: 10.1016/j.atherosclerosis.2018.07.018 30032025

[24] Brunelli N Altamura C Costa CM Altavilla R Palazzo P Maggio P. Carotid artery plaque progression: proposal of a new predictive score and role of carotid intima-media thickness. Int J Environ Res Public Health. (2022) 19:758. doi: 10.3390/ijerph19020758 35055580 PMC8776120

[25] López-Melgar B Fernández-Friera L Oliva B García-Ruiz JM Sánchez-Cabo F Bueno H. Short-term progression of multiterritorial subclinical atherosclerosis. J Am Coll Cardiol. (2020) 75:1617–27. doi: 10.1016/j.jacc.2020.02.026 32273027

[26] Wu TW Chan HL Hung CL Lu IJ Wang SD Wang SW. Differential patterns of effects of age and sex on metabolic syndrome in Taiwan: implication for the inadequate internal consistency of the current criteria. Diabetes Res Clin Pract. (2014) 105:239–44. doi: 10.1016/j.diabres.2014.04.027 24933651

[27] Stein JH Korcarz CE Hurst RT Lonn E Kendall CB Mohler ER. Use of carotid ultrasound to identify subclinical vascular disease and evaluate cardiovascular disease risk: a consensus statement from the American Society of Echocardiography Carotid Intima-Media Thickness Task Force. Endorsed by the Society for Vascular Medicine. J Am Soc Echocardiogr. (2008) 21:93–111. doi: 10.1016/j.echo.2007.11.011 18261694

[28] Touboul PJ Hennerici MG Meairs S Adams H Amarenco P Bornstein N. Mannheim carotid intima-media thickness and plaque consensus (2004-2006-2011). An update on behalf of the advisory board of the 3rd, 4th and 5th watching the risk symposia, at the 13th, 15th and 20th European Stroke Conferences, Mannheim, Germany, 2004, Brussels, Belgium, 2006, and Hamburg, Germany, 2011. Cerebrovasc Dis. (2012) 34:290–6. doi: 10.1159/000343145 PMC3760791 23128470

[29] Rothwell PM Warlow CP. Low risk of ischemic stroke in patients with reduced internal carotid artery lumen diameter distal to severe symptomatic carotid stenosis: cerebral protection due to low poststenotic flow? On behalf of the European Carotid Surgery Trialists’ Collaborative Group. Stroke. (2000) 31:622–30. doi: 10.1161/01.STR.31.3.622 10700495

[30] Chen PC Jeng JS Hsu HC Su TC Chien KL Lee YT. Carotid atherosclerosis progression and risk of cardiovascular events in a community in Taiwan. Sci Rep. (2016) 6:25733. doi: 10.1038/srep25733 27169625 PMC4864369

[31] Li S Tang X Luo Y Wu B Huang Z Li Z. Impact of long-term glucose variability on coronary atherosclerosis progression in patients with type 2 diabetes: a 2.3 year follow-up study. Cardiovasc Diabetol. (2020) 19:146. doi: 10.1186/s12933-020-01126-0 32977802 PMC7517679

[32] Simó R Guerci B Schernthaner G Gallwitz B Rosas-Guzmàn J Dotta F. Long-term changes in cardiovascular risk markers during administration of exenatide twice daily or glimepiride: results from the European exenatide study. Cardiovasc Diabetol. (2015) 14:116. doi: 10.1186/s12933-015-0279-z 26338040 PMC4558893

