# Supplementary material for: Glucose-lowering medications and glucose levels as the major determinants of progression of carotid atherosclerosis in middle-aged adults and elders: a community-based prospective study
Source: Front Endocrinol (Lausanne). 2024 Oct 18;15:1425027. doi: 10.3389/fendo.2024.1425027 (PMC11527687; doi:10.3389/fendo.2024.1425027)
Supplement: Supplementary file 1 [file DataSheet1.pdf]

Supplementary Table. Age-specific progression rates of carotid atherosclerosis in female and male subjects.

Age (years)	Females				Males			
	At risk population (n)	Cumulative incidence (%)			At risk population (n)	Cumulative incidence (%)		
		Either	Plaque number increase	Stenosis increase		Either	Plaque number increase	Stenosis increase
<55	62	32.3	30.6	4.8	53	50.9	47.2	11.3
55-59	87	44.8	43.7	19.5	39	66.7	59.0	25.6
60-64	102	52.0	44.1	19.6	67	61.2	56.7	23.9
65-69	102	55.9	49.0	19.6	83	69.9	66.3	27.7
70-74	30	76.7	70.0	50.0	32	62.5	50.0	25.0
All	383	50.1	45.2	19.6	374	62.8	57.3	23.0
